# Sir Peter Lachmann—Outstanding and Inspirational Immunologist

**DOI:** 10.3390/v13081534

**Published:** 2021-08-03

**Authors:** Ian McConnell

**Affiliations:** Department of Veterinary Medicine, University of Cambridge, Cambridge CB2 1TN, UK; im200@cam.ac.uk; Tel.: +44-07824835308

Sir Peter Lachmann was an exceptional and gifted scientist whose intellectual contributions to biomedical science have been immense. He was an inspirational leader whose scientific and intellectual influence was wide ranging, influential, and went far beyond his field. He was one of the most outstanding immunologists of the last 50 years.

His major scientific discoveries were in the field of complement. In both the innate and adaptive immune response, the complement system plays a key role in host defence against viral, bacterial, fungal, and protozoan pathogens. The complement system is a complex and highly regulated molecular system under exquisite homeostatic control to prevent untoward activation and immunopathology. Through his understanding of the alternative pathway of complement, he elucidated the key role that the third component of complement, C3, and its breakdown fragments played in the early stages of complement activation. His C3 ‘tickover hypothesis’ and the details of the C3b feedback loop proved seminal to understanding the activation and dysregulation of the complement system in inflammatory pathways. This led to the molecular understanding of the key components of both the classical and alternative pathways of complement activation and their role in virus immunity immunopathology. 

Peter’s research career of some 50+ years spanned both the fundamental and clinical aspects of immunology, leading to the publication of more than 450 research papers in top journals. His discoveries were at the cutting edge of immunology. His publications included important discoveries on complement inhibitors, complement genetics, and the genetic deficiencies in human complement that predispose to life-threatening infections. Through the study of complement polymorphisms, his research group and that of his many colleagues defined the genetic linkages between the different complement proteins. This has led to a clearer understanding of the inherited deficiencies of the terminal components of the human complement system in recurrent infections. The recent emergence of the dual role of C3 of the complement system both in coronavirus immunity and the inflammatory cascades in severe coronavirus infections illustrates the central role that the complement system plays in viral infections caused by SARS-CoV, MERS-CoV, and SARS-CoV 2. In due course, the rational design of complement inhibitors based on research leads coming from Peter’s complement research will offer new approaches to controlling the severity of coronavirus diseases. In this context, Sir Peter Lachmann was a biomedical scientist ahead of his time. 

He had a distinctive lecturing style that was memorable and often enlivened scientific meetings. I recall him delivering an immunology lecture on our advanced course in immunology at the Postgraduate Medical School at the Hammersmith Hospital in London on Theories of Antibody Formation. To generate the vast range of antibody and T cell specificities required to deal with the universe of antigens, it had been hypothesised that the immune system required a generator of diversity (GOD). Peter’s view was that as T and B lymphocytes both recognise antigen that there is either one GOD for both cell types—*a monotheistic argument*, or one for T cells and one for B cells—*a ditheistic argument*. At that time (1975), the molecular nature of the T and B cell receptors was unresolved, and Peter’s conclusion was that, in his view, he tended towards ‘*an agnostic ditheism*’.

He was elected Fellow of the Royal Society in 1996 and received many academic accolades including the Gold Medal of the European Complement Network in 1997. He was knighted for service to medical science in 2002.

He has been a huge and constant part of hundreds of scientific lives and careers in science and medicine, including my own. His critical insights and influence have had a wide impact both nationally and internationally. He was always very supportive of younger generations of research scientists. There are many basic and clinical scientists in senior academic positions across the globe who were privileged to have worked with Peter and to have had the support of such a brilliant scientific mind.

Sir Peter was an inspirational leader whose scientific and intellectual influence was colossal and went far beyond his field. He had global stature in biomedical sciences through his research, education, and mentoring of the next generation of scientists. In 1998, he became the founding President of the Academy of Medical Sciences, now the foremost academy of medical sciences in the UK. The Academy has proven to be a much-needed academy. Without his foresight and intellectual drive, the academy would not have come into existence. Currently, the Academy of Medical Sciences broadly impacts the UK biomedical science developments of benefit to Government policies and many research organisations. His critical insights, influence, and wise counsel were always sought after and held in high esteem by many in the scientific community and the pharmaceutical industry. He was President of the Royal College of Pathologists and Vice President and Biological Secretary of the Royal Society. His many roles involved him in ethical and policy controversies in medical and biological science. He was a Fellow of Christ’s College and Honorary Fellow of Trinity College.

I first met Peter when I was a graduate student in 1967 in the laboratory of Professor RRA Coombs, FRS, who had developed the Coombs Test for haemolytic disease of the newborn. Robin Coombs was also the PhD supervisor of Peter. I had modified a sensitive haemolytic plaque assay for the detection of complement by single cells using red cells prepared by Peter and coated with complement components C5, 6, and 7, known as EC567 cells. By incorporating C9 in the plaquing medium, any tissue cells which secreted C8 were detected as plaque-forming cells. Peter told me to be in the lab at 8 a.m. when he had prepared the EC567, and I prepared the cell populations. By midmorning, C8 PFC were detected in the spleen and liver, and I rushed to show Peter the results. Peter was nowhere to be seen. By 6 p.m., he finally reappeared; I asked him where he had been, to which he replied, in his characteristic challenging style, ‘Surely you knew (one of his favourite remarks which always challenged ones knowledge), that when the temperature in the Cambridge falls below freezing for 4 consecutive days in the winter the University declares it a skating holiday! I have been skating in the Fens. It is a well known University Statute—you should look it up in Statutes and Ordinances of the University’. Such was his wonderful encyclopaedic memory and love of obscure facts—he was, however, very interested in my results, thus began a lifetime of knowing and working with Peter (Figure 1). 

Sir Peter was loyal, generous, and supportive to many of his colleagues across the world and leaves a huge legacy. He will be much missed. Our sympathies are with his wife Sylvia and his family. Sylvia was a huge support to Peter and his family. Both Peter and Sylvia hosted many splendid occasions in his lovely house in Cambridge. Visiting scientists who came to Cambridge to work with Peter in his laboratory could always be assured of a warm and intellectually stimulating time.

Ian McConnell.

Emeritus Professor of Veterinary Science, University of Cambridge.

## Figures and Tables

**Figure 1:**
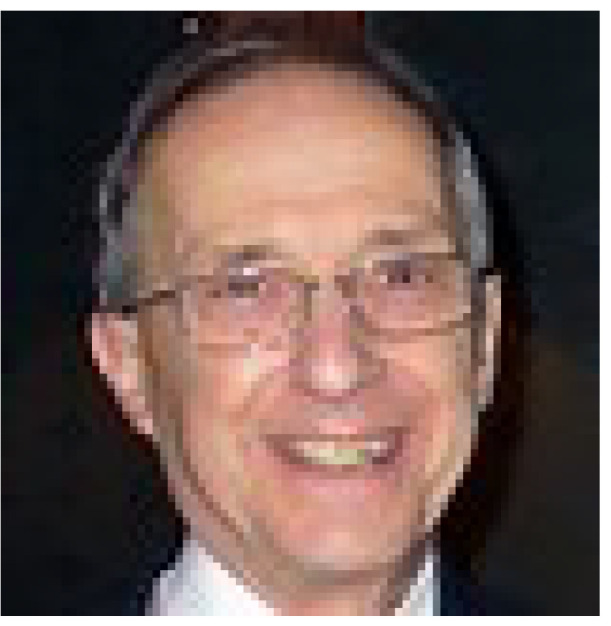
Sir Peter J. Lachmann—a quite rare photo of a laughing Peter.

